# Layer upon layer: Imaging techniques of bladder matrix stone: A case report

**DOI:** 10.1016/j.radcr.2024.08.095

**Published:** 2024-09-17

**Authors:** Carlos Paredes, Blanca Paño, Carmen Sebastià, Bernat Padulles, Leonardo Rodríguez, Jaume Borrell-Vega, Carlos Nicolau

**Affiliations:** a Diagnostic Imaging Center (CDI), Department of Radiogiagnosis, Hospital Clínic, Barcelona, Spain; b Clinic Institute of Nephrology and Urology (ICNU), Department of Urology, Hospital Clínic, Barcelona, Spain; c Center for Biomedical Diagnosis (CDB), Department of Pathology, Hospital Clínic, Barcelona, Spain; d Department of Anesthesia, Resuscitation and Pain Management, Hospital Clínic, Barcelona, Spain

**Keywords:** Matrix lithiasis, Bladder stones, Surgical management, Urology

## Abstract

Matrix lithiasis within the bladder is an extremely rare and diagnostically challenging condition, characterized by its atypical presentation and complex imaging appearance. We report the case of a 69-year-old woman with nonspecific urinary symptoms, including hematuria and abdominal pain. Initial ultrasonography revealed an echogenic lesion on the bladder wall, leading to further investigations using computed tomography (CT) and magnetic resonance imaging (MRI). CT imaging initially delineated the lesion's structure, revealing a complex, multilayered cystic mass. Subsequent MRI provided detailed insights into the internal architecture of the mass, further elucidating its layered structure. Although a parasitic etiology was initially suspected, matrix lithiasis was later confirmed. This case highlights the critical role of a comprehensive imaging strategy in diagnosing rare urological conditions and emphasizes the importance of multimodal imaging in differentiating potential diagnoses.

## Introduction

Roughly 5 percent of urine stones are bladder stones [1]. The percentage of matrix stones in these stones is quite low. Due to their exceptional rarity, matrix stones, which were first reported in 1817 by Marcet et al. have not received much attention in the literature. Three series of cases and a small number of case reports involving urinary matrix have been published too yet. Five cases over a period of 5 years were published by Stoller et al. in 1994. In contrast to the typical urological stones, which are hard and brittle, these patients had soft, malleable, and amorphous stones [2]. Five instances presenting with urinary matrix stones were documented in 2005 by Bani-Hani et al. [3] in a prospective investigation. In 2008, 3 years later, Hemendra et al. [4] examined every patient who had percutaneous nephrolithotomy throughout a 5-year span. A total of 17 patients were found to have matrix calculi at presentation.

Matrix stones are smooth, fibrinous, pliable stones that are also referred to as fibrinomas, colloid calculi, or albumin calculi. Approximately 65% of their dry weight is composed of an amorphous mucopolysaccharide and protein matrix, as opposed to the 25% seen in typical stones [5,6]. Their frequency indicates a possible connection to urinary infections and other underlying disorders, especially in females, diabetics, and individuals who experience repeated UTIs [2,5,7,8]. They share the same risk factors as other forms of lithiasis, including neurogenic bladder and persistent urine flow blockage. Clinical presentation (flank or suprapubic pain, recurrent tract infections, and hematuria) is consistent with that of more typical stones [3].

Matrix stones were found in the renal pelvis area ureter, including the entire renal collecting system, in the majority of the cases that were documented [5]. Only one instance, nevertheless, included matrix calculi in the bladder [6].

## Case report

We present a case of a 69-year-old woman with a medical history significant for hypertension, dyslipidemia, multinodular goitre, cholecystectomy due to cholecystitis, and a long history of type II diabetes. She had been diagnosed with multiple end-organ-failure (retinopathy, polyneuropathy, peripheral angiopathy) due to her suboptimal control of her diabetes mellitus. She had a recent inpatient admission due to acute intestinal ischemia; in March 2021, she underwent an emergency right hemicolectomy under general anesthesia.

She was admitted to the emergency room the on July 2021 with the following urinary symptoms: hematuria with clots and lower abdominal pain. Blood work at the emergency room visit showed no abnormalities, including electrolytes, creatinine, hemoglobin, and white blood count. Urinary sediment showed some leukocytes and erythrocytes. On examination, her vital signs were normal. Physical examination was significant for a well appearing woman with suprapubic and lower abdominal tenderness with no rebound or guarding.

**Fig. 1:**
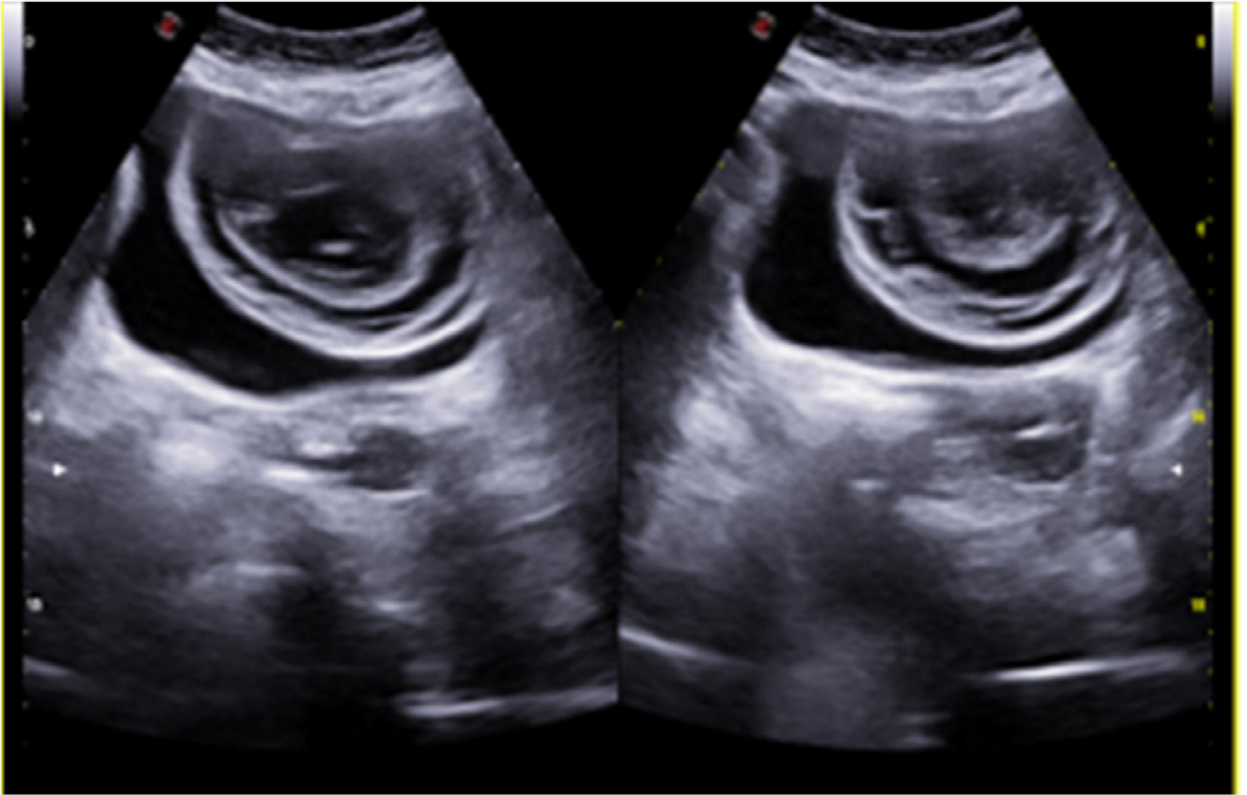
Abdominal ultrasound: Echogenic lesion of approximately 8 cm apparently adhered to the superior bladder wall, with a target-shaped morphology.

**Fig. 2:**
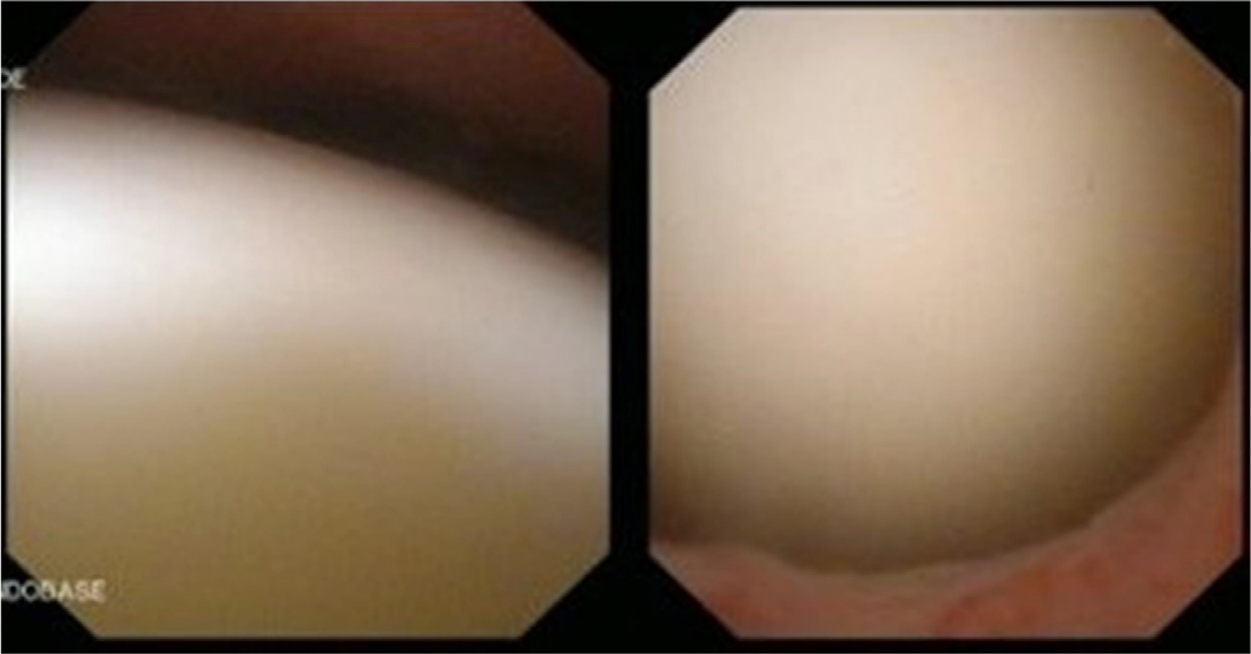
Urethrocystoscopy: Image depicting an oval mass occupying the urinary bladder, not adhered to the bladder mucosa, resembles lithiasis in appearance but is soft to the touch and disintegrates upon contact with the cystoscope.

An abdominal ultrasonography was performed in the first week of August (2 weeks after the symptoms began), to rule out urinary retention and revealed an echogenic lesion of approximately 8 cm apparently adhered to the superior bladder wall, with a target-shaped morphology (Fig. 1). Reviewing anterior imaging tests (Abdominal CT on March 2021), showed no abnormalities at the level of the urinary bladder. Four days later, a urethrocystoscopy was performed, which showed no abnormalities in the urethra and described the urinary bladder as being almost entirely occupied by an oval mass apparently not adhered to the bladder mucosa (Fig. 2).

**Fig. 3:**
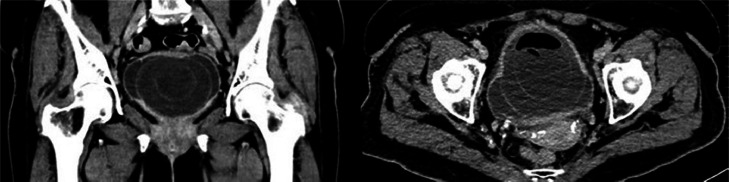
Portal phase abdominal CT: CT images displaying a bladder-occupying lesion with a liquid density core and a thin, homogeneous, nonenhancing wall, 9 cm in diameter. A notable feature is a second cystic lesion within, characterized by a similar wall and a hydroaerial level, indicative of a complex, layered structure.

**Fig. 4:**
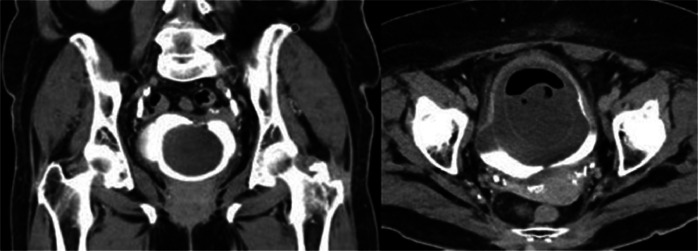
Excretory phase abdominal CT: The excretory phase revealed an endoluminal filling defect at the vesical level corresponding to the previously described bladder mass.

**Fig. 5:**
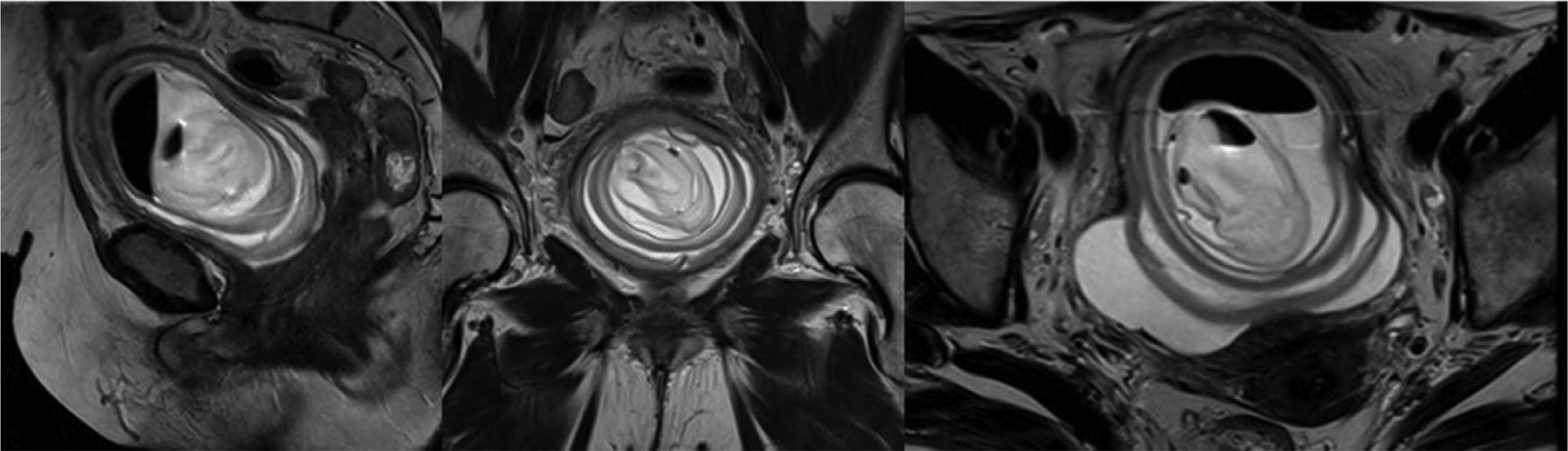
T2 weighted MR scan: MRI scan images showing the bladder walls thickened and grossly hypointense on T2-weighted sequences, suggestive of a chronic inflammatory-infectious process. Multiple layers within the walls of the lesion are arranged concentrically, illustrating a distinct layered configuration.

**Fig. 6:**
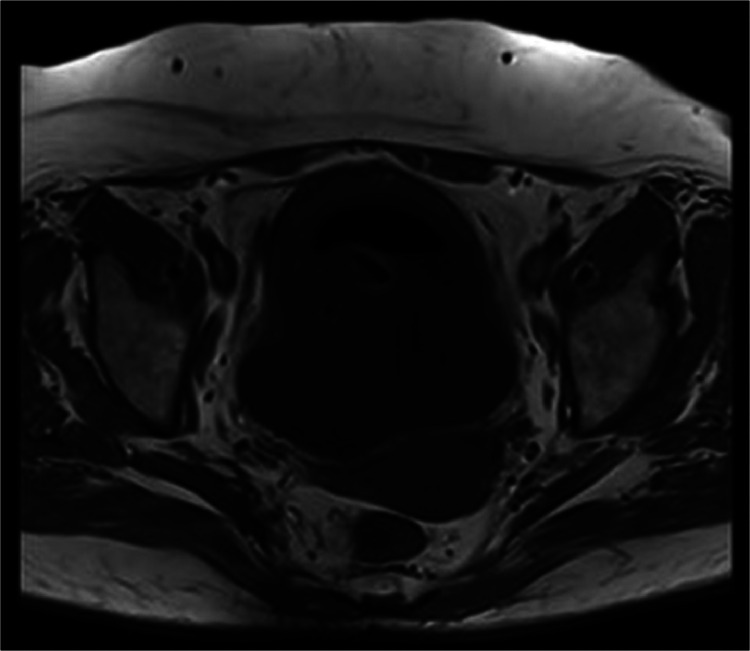
T1 weighted axial MR Scan: Demonstrate no hyperintense areas, indicating the absence of haemorrhagic components.

**Fig. 7:**
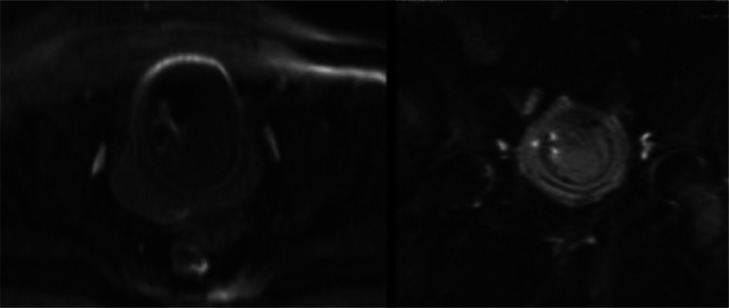
b1000 diffusion-weighted axial and coronal MR Scan: No findings indicative of diffusion restriction areas.

The case was presented to the functional urology committee, and approximately 1 week later computed tomography (CT) scan with intravenous contrast and a magnetic resonance imaging (MRI) scan were performed to enhance lesion characterization and to exclude the involvement of additional organs. The CT showed a bladder unique bladder occupying mass with a second cystic lesion internally (Fig. 3, Fig. 4). The MRI offered a profound insight into the lesions structure (Fig. 5, Fig. 6, Fig. 7).

Initially, the diagnosis was broad. Given the lesion's characteristics (a large endovesical cystic lesion with multiple layers and gas inside), it was suggested that it could be a parasitic lesion, such as hydatidosis, as the most likely option. However, the differential diagnosis also included the presence of foreign bodies and bladder lithiasis.

**Fig. 8:**
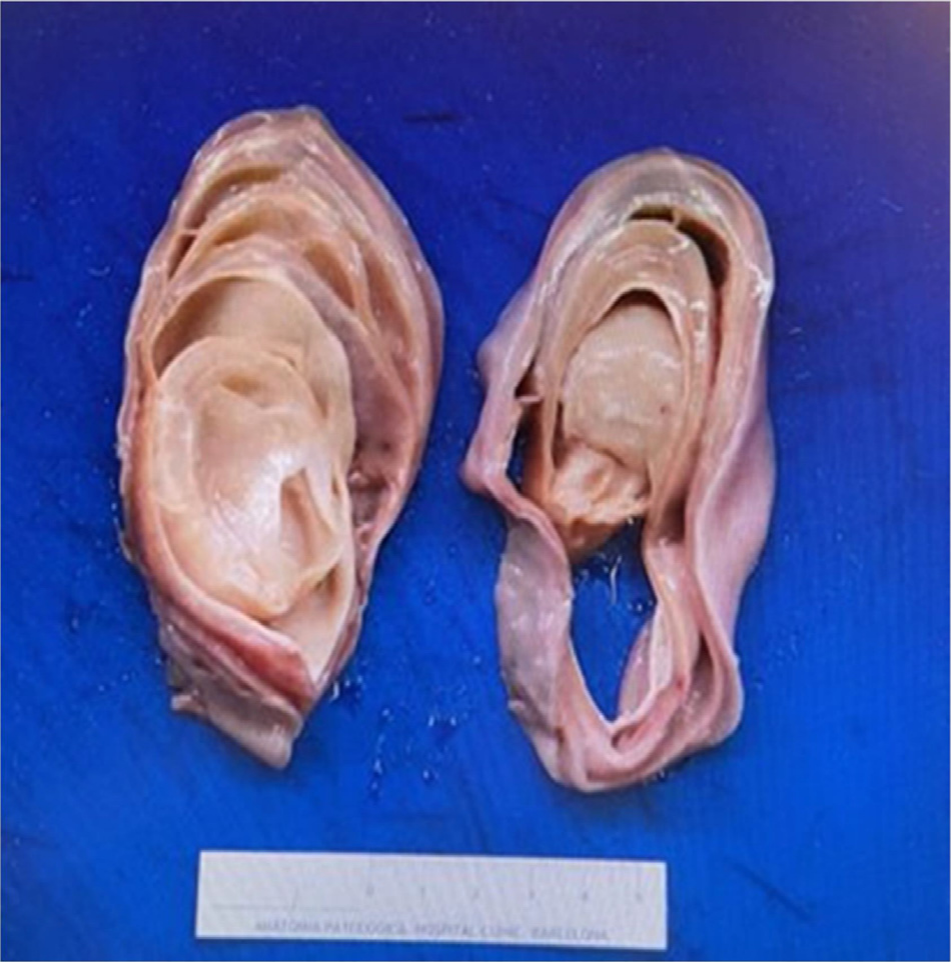
Gross Macroscopic image of the lithiasis. Postoperative image from open suprapubic cystotomy, showcasing a purulent soft mucoproteinaceous mass.

After rediscussing the case with the functional urology committee, the patient eventually underwent an open suprapubic cystotomy for lesion extraction in September. The procedure was performed with no immediate or delayed complications, and surgeons described a purulent soft mucoproteinaceous mass not adherent to any of the bladder walls when mobilized (Fig. 8).

Histopathological analysis showed a lamellar, nonencapsulated lesion formed by thin layers of acellular mucoproteinaceous matrix with a variable number of microorganism colonies (gram-negative bacilli and fungi). No obvious areas of calcification or foreign bodies were observed. The pathologist noted the absence of inflammatory cellularity given the number of bacterial colonies in the sample. Extended spectrum beta-lactamases (ESBLs)-producing Proteus mirabilis was found upon culture of the microorganisms. Subsequently, a definitive diagnosis of intravesical matrix lithiasis was established, underscoring the rare and complex nature of this condition. A course of ertapenem was given postsurgery, leading to the subsidence of symptoms. The patient has not consulted for any other urology-related issues to date.

## Discussion

Matrix stone radiological diagnosis can be challenging, they are typically radiolucent on CT scans and radiolucent on normal abdominal radiographs; they only look amorphous when calcification is present [9]. As a result, the diagnosis is complex.

Furthermore, the radiological differential diagnosis will change based on some characteristics and the location of the lithiasis. When matrix lithiasis are located in the upper excretory tract, it is crucial to distinguish between blood clots, radiolucent urological stones (such as those associated with certain medications, including antiretroviral drugs, ciprofloxazole, sulfonamides, etc.), and detached renal papillary necrosis which affects the upper excretory tract. Since there is no contrast enhancement in tumor lesions, they are not included in the differential diagnosis of matrix lithiasis.

In terms of intravesical localization of matrix lithiasis, the differential diagnosis broadens because these lithiasis can reach larger sizes, as demonstrated in our case (9 cm). In this instance, the primary diagnosis was that of a hydatid cyst, given its radiological appearance closely mirrors that of the described lesions, typically depicted as "spoke in wheel" (multiloculated cystic lesions with a layered appearance that may also present peripheral calcifications). Hydatid disease affecting the urinary tract is rare, and specifically, intravesical localization is extremely uncommon, potentially resulting from the rupture of cysts into the excretory system. The absence of involvement of other organs and the fact that the patient had not been in endemic areas recently were noted, which ultimately led to the exclusion of this diagnosis.

Another diagnostic possibility to consider is that of a foreign body. In the case presented, this was considered because the patient began experiencing symptoms following bladder catheterization, and radiologically, foreign bodies can present with multiple appearances, potentially calcifying if they have been present for an extended period.

Regarding radiological imaging techniques, ultrasound examinations reveal that matrix lithiasis, despite their considerable size, typically do not produce acoustic shadowing. In larger lesions, the layers of lithiasis can be easily discerned via ultrasound, appearing as hyperechoic areas with a linear morphology forming layers. However, they could lead to secondary anatomical alterations that could be visualized, such as dilation of the excretory system.

Unenhanced CT might be considered the gold standard for the diagnosis of matrix stones. First described in 1986 by Sheppard et al. as an “angular filling defect of low attenuation”, there has been some radiological signs that might help clinicians to diagnose this uncommon sort of urinary lithiasis. First, gas inside the matrix stone, especially gas trapped within the various layers of the matrix stone, has been described in some series [6], as we observed in our patient. It has been described as the gas being secondary to active infection and responsible for the laminated stone appearance [10]. This effect, called “onionskin-like calculi”, with layers of 300-700 HU and gas in between, has been consistently described in most of the published series [3,4,11,12]. Upon histological examination, Bommer and colleagues described it as concentric rings of organized matrix with an orderly layered deposition of minerals [12]. Therefore, CT has been postulated as an effective tool to aid in the diagnosis of matrix stones, with specific features that we can use to reveal their location, shape, and internal structure.

The available literature on matrix stones in relation to MRI, is notably sparse. Liu et al. described a hypointense signal in T1-weighted images, with no contrast enhancement following gadolinium administration, and hyperintense signal in T2-weighted images [13]. In our experience, MRI provides additional information compared to CT in several aspects. It has the capability to visualize layers within the walls described in CT, offering a more detailed insight into the structure. Additionally, it facilitates the visualization of a greater number of layered lesions that were not evident on the CT. Overall, MRI offers more profound insights into the already depicted pattern of” onionskin-like calculi."

Conventional open cystolithotomy (CL) has long been regarded as the gold-standard surgical treatment for addressing bladder stones as it has a high stone-free rate (SFR) and long-term published data available,. However new developments in endoscopic and minimally invasive technologies have strengthened the urologist's toolkit for treating bladder stones permanently. Alternatives to CL that may be practical and secure include transurethral cystolithotripsy (TUCL), percutaneous cystolithotripsy (PCCL), extracorporeal shock wave lithotripsy (ESWL), and laparoscopic cystolithotomy (LapCL). It's uncertain if these minimally invasive methods result in worse SFRs, although they might be less morbid, require fewer inpatient stays, and require urinary catheterization than CL. Only stones that exhibit a significant mineral composition can still benefit from extracorporeal shock wave lithotripsy [[14], [15], [16]].

Additionally, prophylactic antibiotic use is advised to reduce the likelihood of asymptomatic UTIs and bacteriuria. Close monitoring including routine urological exams with urine and ultrasound every 3 months is advised in order to enable timely intervention in cases of recurrent lower-size stones by minimally invasive surgical techniques.

In the case presented, given the dimensions of the matrix stone measuring 9 cm, open surgery via a suprapubic cystotomy was deemed the most appropriate approach.

## Conclusion

The diagnosis of matrix stones presents a notable challenge in contemporary clinical practice. However, in the specific case outlined herein, a comprehensive diagnostic approach utilizing bedside ultrasound, urethrocystoscopy, and computed tomography facilitated the precise identification and characterization of the bladder lesion. The distinctive layered appearance observed on computed tomography, further corroborated by MRI, underscores the invaluable contribution of these imaging modalities to the accurate diagnosis of matrix stones. Despite the scarcity of literature on matrix stones and MRI, our findings underscore the significance of this diagnostic modality in providing deeper insights into the structure and composition of such lesions. Continued research efforts are imperative to further refine our understanding and management strategies for matrix stones in clinical practice. Given the rarity of this pathology, clinicians must maintain a heightened level of suspicion to ensure prompt diagnosis and intervention when warranted.

## Patient consent

Written, informed consent for the publication of this case has been obtained from the patient. This consent includes the use of personal health information and any potentially identifiable details within the context of this publication
